# Database Resources of the National Genomics Data Center, China National Center for Bioinformation in 2024

**DOI:** 10.1093/nar/gkad1078

**Published:** 2023-11-29

**Authors:** CNCB-NGDC Members and Partners, Xue Bai, Yiming Bao, Shaoqi Bei, Congfan Bu, Ruifang Cao, Yongrong Cao, Hui Cen, Jinquan Chao, Fei Chen, Huanxin Chen, Kai Chen, Meili Chen, Miaomiao Chen, Ming Chen, Qiancheng Chen, Runsheng Chen, Shuo Chen, Tingting Chen, Xiaoning Chen, Xu Chen, Yuanyuan Cheng, Yuan Chu, Qinghua Cui, Lili Dong, Zhenglin Du, Guangya Duan, Shaohua Fan, Zhuojing Fan, Xiangdong Fang, Zhanjie Fang, Zihao Feng, Shanshan Fu, Feng Gao, Ge Gao, Hao Gao, Wenxing Gao, Xiaoxuan Gao, Xin Gao, Xinxin Gao, Jiao Gong, Jing Gong, Yujie Gou, Siyu Gu, An-Yuan Guo, Guoji Guo, Xutong Guo, Cheng Han, Di Hao, Lili Hao, Qinwen He, Shuang He, Shunmin He, Weijuan Hu, Kaiyao Huang, Tianhao Huang, Xinhe Huang, Yuting Huang, Peilin Jia, Yaokai Jia, Chuanqi Jiang, Meiye Jiang, Shuai Jiang, Tao Jiang, Xiaoyuan Jiang, Enhui Jin, Weiwei Jin, Hailong Kang, Hongen Kang, Demian Kong, Li Lan, Wenyan Lei, Chuan-Yun Li, Cuidan Li, Cuiping Li, Hao Li, Jiaming Li, Jiang Li, Lun Li, Pan Li, Rujiao Li, Xia Li, Yanyan Li, Yixue Li, Zhao Li, Xingyu Liao, Shiqi Lin, Yihao Lin, Yunchao Ling, Bo Liu, Chun-Jie Liu, Dan Liu, Guang-Hui Liu, Lin Liu, Shulin Liu, Wan Liu, Xiaonan Liu, Xinxuan Liu, Yiyun Liu, Yucheng Liu, Mingming Lu, Tianyi Lu, Hao Luo, Huaxia Luo, Mei Luo, Shuai Luo, XiaoTong Luo, Lina Ma, Yingke Ma, Jialin Mai, Jiayue Meng, Xianwen Meng, Yuanguang Meng, Yuyan Meng, Wei Miao, Ya-Ru Miao, Lingbin Ni, Zhi Nie, Guangyi Niu, Xiaohui Niu, Yiwei Niu, Rong Pan, Siyu Pan, Di Peng, Jianzhen Peng, Juntian Qi, Yue Qi, Qiheng Qian, Yuxin Qin, Hongzhu Qu, Jian Ren, Jie Ren, Zhengqi Sang, Kang Shang, Wen-Kang Shen, Yanting Shen, Yirong Shi, Shuhui Song, Tingrui Song, Tianhan Su, Jiani Sun, Yanlin Sun, Yanling Sun, Yubin Sun, Bixia Tang, Dachao Tang, Qing Tang, Zhixin Tang, Dongmei Tian, Feng Tian, Weimin Tian, Zhixi Tian, Anke Wang, Guangying Wang, Guoliang Wang, Jianxin Wang, Jie Wang, Peihan Wang, Pengyu Wang, Wenquan Wang, Yanqing Wang, Yibo Wang, Yimin Wang, Yonggang Wang, Zhonghuang Wang, Haobin Wei, Yuxiang Wei, Zhiyao Wei, Dingfeng Wu, Gangao Wu, Sicheng Wu, Song Wu, Wanying Wu, Wenyi Wu, Zhile Wu, Zhiqiang Xia, Jingfa Xiao, Leming Xiao, Yun Xiao, Guiyan Xie, Gui-Yan Xie, Jianbo Xie, Yubin Xie, Jie Xiong, Zhuang Xiong, Danyang Xu, Shuhua Xu, Tianyi Xu, Tingjun Xu, Yongbiao Xue, Yu Xue, Chenghao Yan, Dechang Yang, Fangdian Yang, Fei Yang, Hongwei Yang, Jian Yang, Kuan Yang, Nan Yang, Qing-Yong Yang, Sen Yang, Xiaoyu Yang, Xiaoyue Yang, Xilan Yang, Yun-Gui Yang, Weidong Ye, Caixia Yu, Fudong Yu, Shuhuan Yu, Chunhui Yuan, Hao Yuan, Jingyao Zeng, Shuang Zhai, Chi Zhang, Feng Zhang, Guoqing Zhang, Mochen Zhang, Peng Zhang, Qiong Zhang, Rongqin Zhang, Sisi Zhang, Wanyu Zhang, Weiqi Zhang, Weizhi Zhang, Xin Zhang, Xinxin Zhang, Yadong Zhang, Yang Zhang, Yiran Zhang, Yong E Zhang, Yuansheng Zhang, Zhang Zhang, Zhe Zhang, Dongli Zhao, Fangqing Zhao, Guoping Zhao, Miaoying Zhao, Wei Zhao, Wenming Zhao, Xuetong Zhao, Yilin Zhao, Yongbing Zhao, Zheng Zhao, Xinchang Zheng, Yu Zheng, Chenfen Zhou, Haokui Zhou, Xincheng Zhou, Xinyu Zhou, Yincong Zhou, Yubo Zhou, Junwei Zhu, Lixin Zhu, Ruixin Zhu, Tongtong Zhu, Wenting Zong, Dong Zou, Zhixiang Zuo

## Abstract

The National Genomics Data Center (NGDC), which is a part of the China National Center for Bioinformation (CNCB), provides a family of database resources to support the global academic and industrial communities. With the rapid accumulation of multi-omics data at an unprecedented pace, CNCB-NGDC continuously expands and updates core database resources through big data archiving, integrative analysis and value-added curation. Importantly, NGDC collaborates closely with major international databases and initiatives to ensure seamless data exchange and interoperability. Over the past year, significant efforts have been dedicated to integrating diverse omics data, synthesizing expanding knowledge, developing new resources, and upgrading major existing resources. Particularly, several database resources are newly developed for the biodiversity of protists (P10K), bacteria (NTM-DB, MPA) as well as plant (PPGR, SoyOmics, PlantPan) and disease/trait association (CROST, HervD Atlas, HALL, MACdb, BioKA, BioKA, RePoS, PGG.SV, NAFLDkb). All the resources and services are publicly accessible at https://ngdc.cncb.ac.cn.

## Graphical Abstract

**Graphical Abstract:**
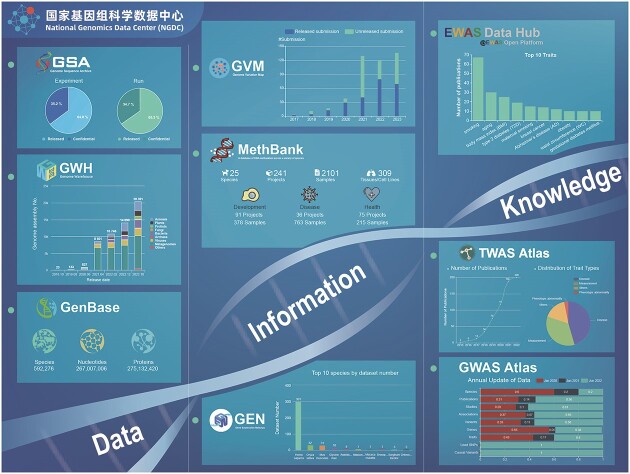

## Introduction

The National Genomics Data Center (NGDC) is affiliated to Beijing Institute of Genomics (BIG), Chinese Academy of Sciences (CAS), and China National Center for Bioinformation (CNCB) (1). Established in 2019, CNCB-NGDC has collaborated with CAS institutions, viz., Institute of Biophysics and Shanghai Institute of Nutrition and Health, as well as formed partnerships with other organizations (https://ngdc.cncb.ac.cn/partners). Over the last decades, advancements in high-throughput technologies have enabled researchers to simultaneously analyze multiple layers of biological information with unprecedented speed and accuracy. Large-scale high-throughput sequencing projects have been conducted globally to study the genetic basis of diseases and unravel complex biological processes (2,3). Projects like the 1000 Genomes Project (2), the Cancer Genome Atlas (3), and the UK BioBank (4) have contributed to the generation of extensive genomic datasets from diverse populations and disease cohorts. These datasets have provided invaluable resources for studying genetic variations, identifying disease-associated genes, and exploring molecular mechanisms underlying complex diseases. Moreover, single-cell sequencing technologies have emerged as powerful tools to study cellular heterogeneity (5), developmental processes (6), disease mechanisms (7), and complex biological systems (8) with unprecedented resolution (9). In particular, spatial transcriptomics techniques capture the spatial information of gene expression patterns and offer a deeper understanding of tissue architecture, cell-to-cell communication, and tumor heterogeneity (10). As a result, an immense amount of multi-omics data has been generated at an ever-increasing rate and scale, necessitating the development of resources that facilitate data synthesizing, interoperability and sharing.

**Figure 1.**
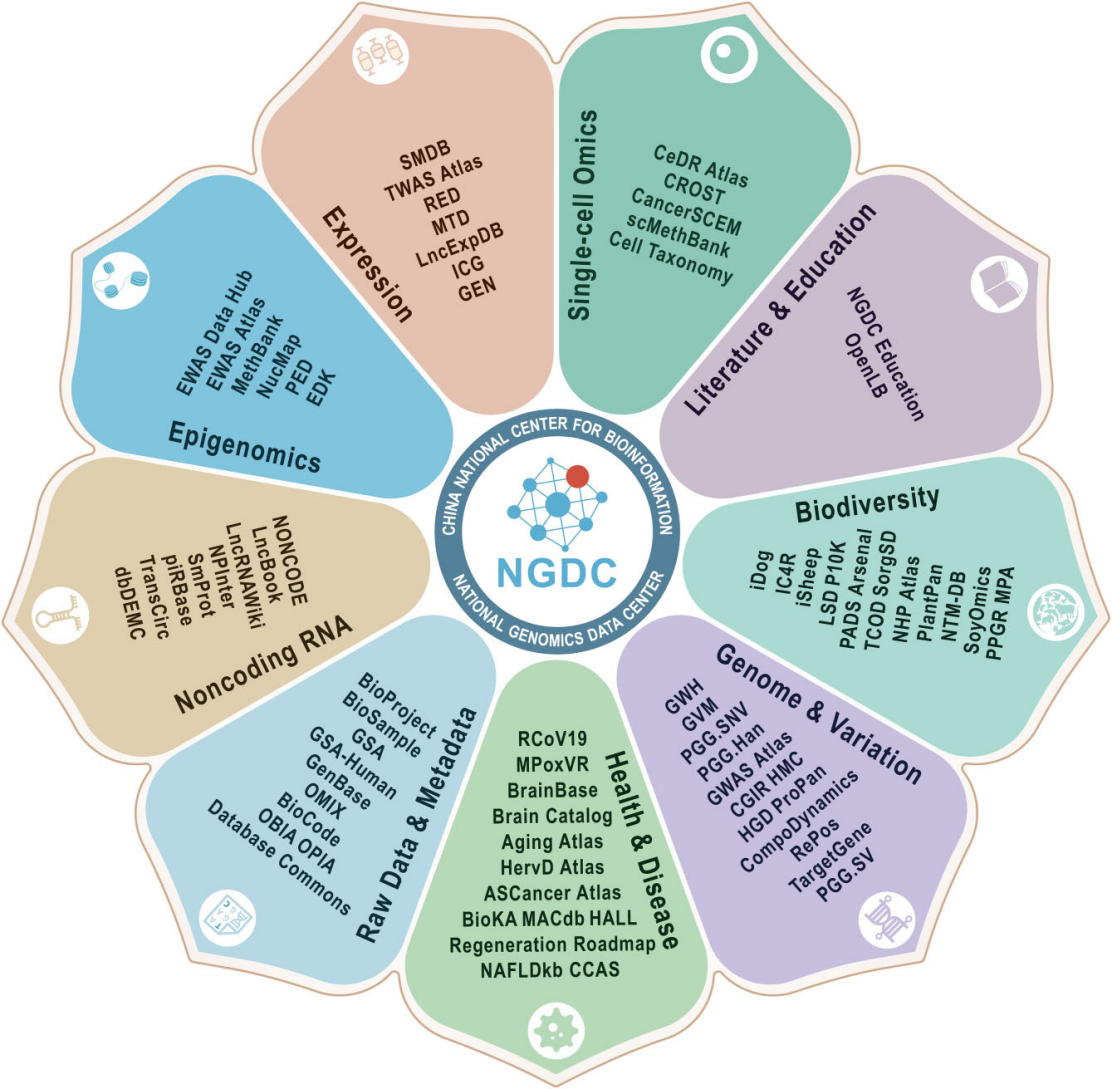
The core database resources of CNCB-NGDC organized into various categories. These database resources are publicly accessible and searchable through CNCB-NGDC home page at https://ngdc.cncb.ac.cn. A full list of data resources is shown at https://ngdc.cncb.ac.cn/databases.

**Figure 2.**
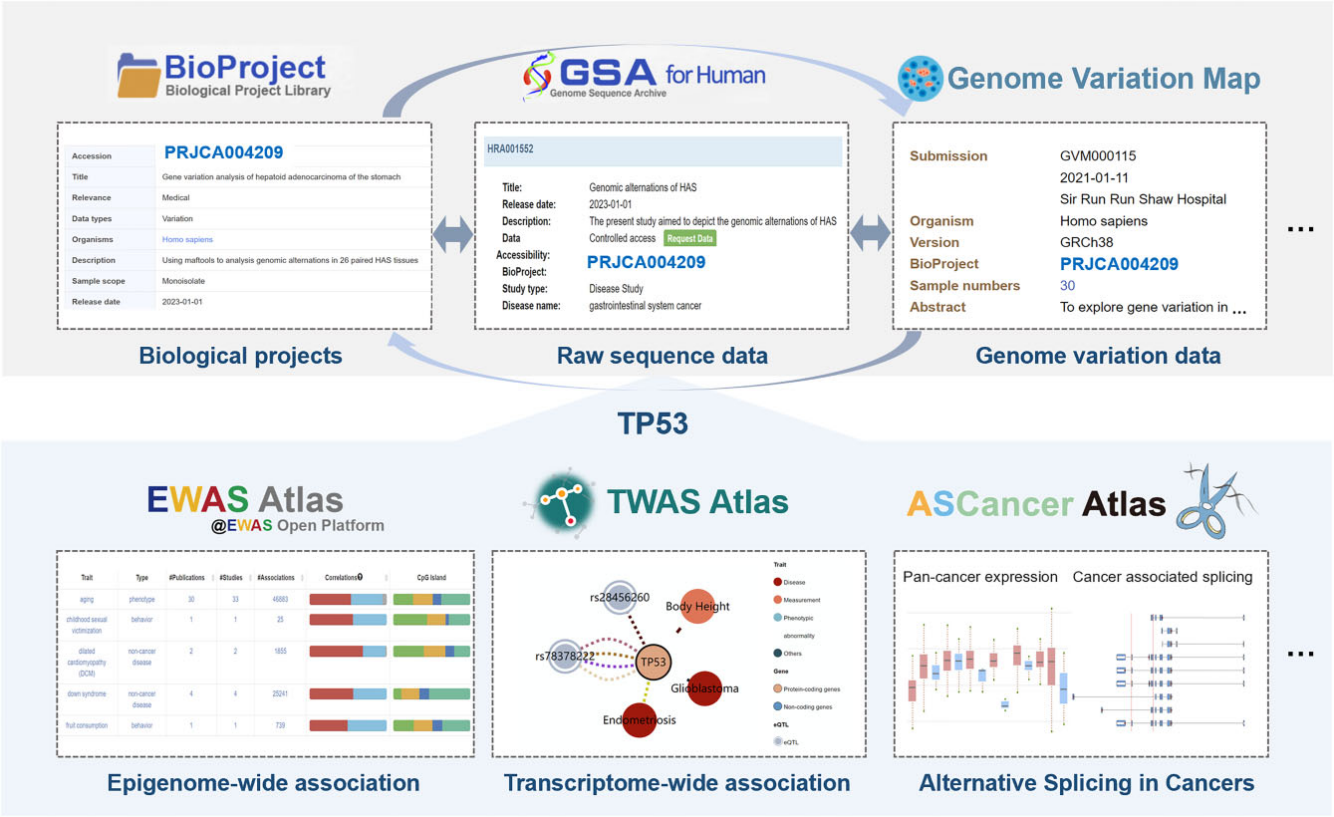
The connectivity of CNCB-NGDC core databases. BioProject, GSA-human and GVM are closely interconnected through a BioProject ID (e.g. PRJCA004209), allowing users to easily navigate between databases and access related information including biological project (https://ngdc.cncb.ac.cn/bioproject/browse/PRJCA004209), genomic information (https://ngdc.cncb.ac.cn/gsa-human/browse/HRA001552) and genetic variation (https://ngdc.cncb.ac.cn/gvm/getProjectDetail?project=GVM000115). Based on these information, users can further find a wealth of knowledge about any specific gene, taking TP53 for example, such as its epigenetic associations in EWAS Atlas (https://ngdc.cncb.ac.cn/ewas/browse?gene=TP53), transcriptional associations in TWAS Atlas (https://ngdc.cncb.ac.cn/twas/genedetail/ENSG00000141510.16), and cancer-associated splicing events in ASCancer Atlas (https://ngdc.cncb.ac.cn/ascancer/search?genename=TP53).

With the rapid growth of large-scale high-throughput sequencing projects globally, CNCB-NGDC serves as a central hub for the collection, integration and curation of diverse genomics datasets. In the past year, CNCB-NGDC has been dedicated to the development of new resources and the continuous updating of existing resources, aiming to provide open access to a family of resources for advancing life and health sciences globally (11–22). Importantly, several core database resources have been recommended by major publishers, which has greatly facilitated the efficient deposition and open sharing of biomedical data. Furthermore, CNCB-NGDC has established close collaborations with the International Nucleotide Sequence Database Collaboration (INSDC) (23) by mirroring the metadata and sequence data from NCBI SRA (Sequence Read Archive) (24). In this article, we provide a brief overview of new developments and recent updates in CNCB-NGDC, highlighting its core resources and services (Figure 1). Importantly, CNCB-NGDC databases are highly interconnected, forming a comprehensive network that allows users to seamlessly navigate between databases, access relevant information, and conduct comprehensive studies (Figure 2). All these resources and services play a crucial role in supporting research and are publicly available on the CNCB-NGDC homepage (https://ngdc.cncb.ac.cn).

## New developments

### Raw data & metadata

#### GenBase

GenBase (https://ngdc.cncb.ac.cn/genbase) is an open-access data repository dedicated to archiving, searching, and sharing nucleotide sequences. It accepts various data submissions, including mRNA, genomic DNA and ncRNA as well as small genomes like organelles, viruses, plasmids and phages. GenBase provides a user-friendly bilingual submission portal with automatic validation and manual curation. Its standardized data structures and quality control procedures are compatible with those of GenBank (25), enabling seamless data exchange with the INSDC (23). GenBase incorporates all sequences from GenBank with daily updates, currently housing 265 969 760 nucleotide and 268 933 169 protein sequences. Meanwhile, it has received a total of 1103 direct submissions as of 14 August 2023, including 37 981 nucleotide sequences and 362 296 annotated protein sequences across 138 species. Of these, 34 477 nucleotide sequences (91%) and 340 491 annotated protein sequences (94%) have been released and are publicly accessible. Particularly, GenBase has received and released 31 312 SARS-CoV-2 genome sequences with standardized annotations. In summary, GenBase is a critical resource for archiving and incorporating a large variety of nucleotide sequence data, offering free and public data services to support worldwide research activities.

#### OBIA

The Open Biomedical Imaging Archive (OBIA; https://ngdc.cncb.ac.cn/obia) serves as a repository for archiving biomedical images and associated clinical data (26). OBIA adopts five data objects (Collection, Individual, Study, Series, and Image) for data organization and accepts submissions of biomedical images from all over the world. To ensure data privacy, OBIA has established a standardized de-identification and quality control process and offered two types of data accessibility: open access and controlled access. As of August 2023, OBIA has housed 937 individuals, 4136 studies, 24 701 series and 1 938 309 images covering 9 modalities and 30 anatomical sites. OBIA differentiates itself from other related databases by providing imaging data of various modalities, anatomical sites, and diseases in a common DICOM format. In addition, OBIA supports both metadata retrieval and image retrieval. Importantly, OBIA establishes internal links with NGDC’s BioProject accessions and individual accessions in GSA-Human, facilitating users to easily obtain not only biomedical images, clinical data but also multi-omics data.

#### OPIA

The Open Plant Image Archive (OPIA, https://ngdc.cncb.ac.cn/opia/) is an open archive of plant images and phenotypic traits (i-traits) derived from high-throughput phenotyping platforms (27). Currently, OPIA houses 56 datasets across 11 plants, comprising a total of 566 225 images with 2 417 186 labeled instances. It also incorporates 56 image-based i-traits derived from 18 644 individual RGB images across 3 datasets. These i-traits are annotated using the Plant Phenotype and Trait Ontology (PPTO) and cross-linked with GWAS Atlas. Additionally, each dataset in OPIA is assigned an evaluation score that considers factors such as image data volume, image resolution, and the number of labeled instances. OPIA also provides useful tools for online image pre-processing and submission. Collectively, OPIA provides open access to valuable datasets and phenotypic traits across diverse plants and thus bears great potential to play a crucial role in facilitating artificial intelligence-assisted breeding research.

### Single-cell omics

#### CROST

CROST (https://ngdc.cncb.ac.cn/crost) is a comprehensive repository of spatial transcriptomics. It contains 182 spatial transcriptomic datasets comprising 1033 high-quality samples from 5 technology platforms, 8 species and 56 diseases (28). A total of 48 043 tumor-related spatially variable genes (SVGs) are identified across these datasets. Additionally, it includes a standardized spatial transcriptome data processing pipeline, integrates deconvolution spatial transcriptomics data, and performs correlation, colocalization, intercellular communication and biological function annotation analyses. Moreover, CROST integrates transcriptomic, epigenomic, and genomic data to investigate tumor-associated SVGs, providing a comprehensive insight into their roles in cancer progression and prognosis. Furthermore, CROST provides two online tools: single-sample gene set enrichment analysis (ssGSEA) and SpatialAP, enabling users to annotate and analyze uploaded spatial transcriptomics data. Collectively, CROST offers fresh and comprehensive insights into tissue structure and serves as a foundation for understanding multiple biological mechanisms in diseases, particularly in tumor tissues.

### Expression

#### SMDB

The SMDB (https://www.biosino.org/smdb) (29) is an essential database that facilitates the exploration and understanding of spatial transcriptomics (ST) data comprehensively and interactively. Its multimodal integration and customisable workspaces offer researchers a powerful and versatile platform to investigate the intricate relationship between spatial data and biological function. In 2D, SMDB enables segmenting slices and identifying gene expression boundaries. Researchers can analyze tissue composition using loaded images and molecular clusters. In 3D, researchers can filter spots based on their specific requirements and reconstruct morphological visualizations. SMDB also provides customizable workspaces that allow for interactive exploration. SMDB includes the pre-loaded Allen Mouse Brain Common Coordinate Framework (CCFv3) from the renowned Allen Institute that serves as a valuable reference for studying the mouse brain, providing researchers with quick access to relevant information.

### Health and disease

#### HervD Atlas

HervD Atlas (https://ngdc.cncb.ac.cn/hervd/) is a knowledgebase integrating Human endogenous retroviruses (HERV)-disease associations curated from numerous publications (30). Currently, HervD Atlas collects 57 253 curated HERV-disease associations from 238 publications, covering 19 274 HERVs (including 18 535 HERV-Terms and 739 HERV-Elements) belonging to six types. The knowledgebase also encompasses 148 ontological diseases grouped into 14 categories and 605 affected or related genes. It features an interactive knowledge graph that visually represents the relationship networks of HERV-disease associations and corresponding genes, enabling researchers to access and explore data of interest efficiently. HervD Atlas serves as a valuable resource and powerful platform with comprehensive HERV-disease knowledge, facilitating our understanding of HERV-disease associations and the development of HERVs as novel diagnostic and therapeutic strategies.

#### HALL

HALL (Human Aging and Longevity Landscape; https://ngdc.cncb.ac.cn/hall/) is a dedicated database centering on the study of human aging and longevity (31). It offers a specialized and comprehensive collection of multi-dimensional datasets derived from various human cohorts. HALL integrates 170 cohorts from 23 countries/regions, including 1913 SNPs, 38 tissue/cell types and over 4 800 000 individuals, ranging from 1 to 119 years and with 59 cohorts including centenarians. HALL features a genome browser with 485 512 epigenomics probes, providing insights into age-related methylation changes. The transcriptome of 5261 age-variant genes has been curated involving a total of 3188 human subjects across 13 tissues. HALL was built upon the foundation of the Aging Biomarker Consortium (ABC). Its comprehensive framework for monitoring age-related changes serves as a platform for developing new markers, diagnostic tools, and strategies to address aging and age-related conditions.

#### MACdb

MACdb (https://ngdc.cncb.ac.cn/macdb/) is a curated knowledgebase of metabolic associations between metabolites and cancers (32). In the current implementation, MACdb has integrated 40 710 cancer-metabolite associations, encompassing 267 traits from 17 categories of cancers with high incidence or mortality. These associations are derived through meticulous manual curation of 1127 studies published in 462 publications. MACdb provides user-friendly browsing functions that allow the exploration of associations across multiple dimensions, such as metabolite, trait, study, and publication. Additionally, it constructs a knowledge graph to present an overall landscape of the relationships among cancer, trait, and metabolite. Furthermore, MACdb offers tools of NameToCid, which maps metabolite names to PubChem CIDs, and Enrichment tools, which aid in enriching the associations of metabolites with various cancer types and traits. MACdb represents an informative and practical resource for evaluating cancer-metabolite associations, with the potential to accelerate hypothesis generation and research on cancer metabolism.

#### NAFLDkb

NAFLDkb (https://www.biosino.org/nafldkb) is a specialized knowledge base and platform for computer-aided drug design against non-alcoholic fatty liver disease (NAFLD) (33). NAFLD incorporates multi-perspective information from public resources including source data, background knowledge and candidate library. The source data includes 40 433 research articles and 1001 clinical trials. The background knowledge consists of 581 investigational drugs, 17 therapeutic strategies, 45 therapeutic targets, 17 associated diseases, 8 records of pathogenesis and 68 *in vitro* and *in vivo* models of NAFLD. The candidate library consists of 1608 repositioning candidates, 147 604 bioactive compounds, 34 419 CMap candidates and 17 704 natural products for NAFLD drug development. The relationships among drug-related entities are presented with knowledge graphs, and AI-powered tools provide chemical structure search, drug-likeness screening, knowledge-based repositioning, and research article annotation.

#### BioKA

BioKA (https://ngdc.cncb.ac.cn/bioka) is a comprehensive disease/trait biomarker (34–37) knowledgebase for animals, including model and domestic animals as well as humans (38). We curate biomarkers and integrate various annotations, such as Gene Ontology terms (GOs), protein structures, protein-protein interaction networks, miRNA targets, metabolism details, expressions, variations, and homologous genes, into a single web platform. BioKA enables cross-species research and offers free public data services for browsing, retrieval, comparison, and downloading. Currently, BioKA houses 16 296 biomarkers associated with 951 mapped diseases/traits across 31 species from 4747 references. These include 11 925 gene/protein biomarkers, 1784 miRNA biomarkers, 1043 mutation biomarkers, 773 metabolic biomarkers, 357 circRNA biomarkers and 127 lncRNA biomarkers. Furthermore, BioKA constructs an interactive knowledge network of biomarkers that includes 7320 entities and 401 208 links across 10 species. Moreover, BioKA provides detailed information on 308 breeds/strains of 13 species and homologous annotations for 8784 biomarkers across 16 species, and offers three online application tools. In summary, BioKA advances human disease research, contributes to understanding animal diseases, and supports livestock breeding.

### Genome and variation

#### RePoS

RePoS (Recent Positive Selection, http://bigdata.ibp.ac.cn/RePoS/) is a newly developed database that integrates and presents recent positive selection signal data for both Chinese and worldwide populations. This database aims to enhance our understanding of genes and traits that have undergone positive selection during human evolution, providing insights into our history and diseases that continue to plague us today. RePoS investigates the multi-population selection footprints of genomic sequences using SDS (39) and iHS (40) data such as NyuWa WGS (41,42), TOPMed (43), 1KGP (44) and UK10K (39) and elucidate phenotypic evolution associated with genomic signatures for both monogenic and polygenic traits. A total of 22.7 million non-redundant variants from five datasets were integrated. In summary, RePoS is designed to facilitate the study of human evolution and phenotype adaptation in global populations.

#### TargetGene

TargetGene (https://ngdc.cncb.ac.cn/targetgene/) is a comprehensive resource of target genes for human genetic variants (45). It establishes connections between genetic variants and their target genes using multiple analytical tools, such as chromatin co-accessibility, 3D interaction, enhancer activities, and quantitative trait loci. The resource includes curated multi-omics data from single-cell and bulk levels, encompassing various human tissues, cell types, developmental stages, and over a thousand genome-wide association studies (GWAS) datasets. Currently, TargetGene comprises 23 838 target genes in 45 tissues and 539 cell types inferred for 574 279 trait-associated genetic variants from 1276 GWAS datasets for various diseases. TargetGene provides user-friendly web interfaces to help users systematically identify and prioritize trait-associated target genes. In summary, TargetGene serves as a valuable resource for understanding the genetic mechanisms behind complex diseases and identifying potential drug targets.

#### PGG.SV

PGG.SV (https://www.biosino.org/pggsv) is a pioneering database leveraging next-generation and third-generation whole-genome sequencing technologies (46). The current version of PGG.SV encompasses a vast dataset of 584 277 structural variations (SVs) from 6048 samples, including 1030 long-read sequenced genomes from 177 global populations. Notably, PGG.SV offers high-quality, fine-scale SVs mapped to both GRCh37 and GRCh38 human reference genomes. This includes previously underrepresented SVs that were difficult to detect using conventional sequencing and microarray data. The database features hierarchical estimates of SV prevalence across diverse geographical populations and offers valuable annotations of SV-related genes, putative functions, and clinical implications. Moreover, it provides an easy-to-navigate interface and offers robust visualization tools for genome-wide SV mapping.

### Biodiversity

#### PlantPan

PlantPan (https://ngdc.cncb.ac.cn/plantpan/) is a comprehensive database containing pan-genome analysis results of 195 genomes from 11 plant species. PlantPan offers detailed insights across five categories: species, genes, gene clusters, genomic variances and genome synteny. PlantPan includes nine graph pan-genomes, 9 127 208 genes, 694 191 gene groups, 413 000 124 genomic variations, 1 616 089 genomic variation groups, 3 345 098 genome synteny and 177 827 genome synteny groups. Each gene group is assigned functional annotations, such as GO annotation, protein functional domains, 23 types of KEGG pathways, 58 types of transcription factors, organic and inorganic resistance, and homologous genes in other species. In summary, PlantPan serves as an invaluable resource for enhancing the utilization of plant pan-genomes in molecular breeding and evolutionary studies.

#### NTM-DB

NTM-DB (Non-Tuberculosis Mycobacteria Database; https://ngdc.cncb.ac.cn/ntmdb) is a public database that integrates the most comprehensive collection of genomic and bioinformatics resources for non-tuberculosis mycobacteria (NTM). It includes a total of 12 748 newly assembled whole-genomes and 3335 GenBank/RefSeq assemblies, covering 177 out of 190 NTM species. Notably, NTM-DB incorporates 705 MLSTs (Multi-Locus Sequence Typing), consisting of 189 type strain genomes (representing 177 species and 12 subspecies) and 181 representative genomes. The database also encompasses 33 240 drug-resistance genes, 7152 drug susceptibility tests, and 74 315 virulence genes. Furthermore, NTM-DB offers an online analytical platform for genotyping, drug-resistance and virulence gene annotation, as well as pan-genomic and phylogenetic analyses. Together, NTM-DB is a comprehensive and innovative platform for the NTM research community, with the potential to assist clinicians in diagnosing and treating various NTM-related diseases.

#### SoyOmics

SoyOmics (https://ngdc.cncb.ac.cn/soyomics) is an integrated multi-omics database for soybean designed to provide a one-stop solution for big data mining (47). The current implementation features comprehensive integration of high-quality omics data, including assembly genomes, graph pan-genome, phenotypic data of representative germplasms, transcriptomic and epigenomic data from different tissues, organs, and accessions, as well as knowledge of quantitative trait locus and genome-wide association study (GWAS). In addition, several commonly easy-to-use toolkits are also equipped for sequence alignment (BLAST), quick-start GWAS analysis (easyGWAS), gene expression pattern analysis (ExpPattern), haplotype analysis (HapSnap), genome position transformation (VersionMap), and sequence extraction (SeqFetch). More importantly, a module named SoyArray is developed to compare divergent sites between two germplasms, which is helpful for parent selection in genetic or breeding studies. Taken together, SoyOmics is of great utility to facilitate deep mining ranging from fundamental research to molecular breeding.

#### The P10K database

The P10K Database (https://ngdc.cncb.ac.cn/p10k/) is a data portal for the Protist 10 000 Genomes Project (P10K). This project was established to address the limited availability of published genomes for protist species, which play significant roles in the biosphere as diverse microscopic eukaryotic organisms separate from fungi, animals, and plants (48). The resulting P10K database serves as a comprehensive platform, compiling and disseminating genome sequences and annotations from various protist groups. Currently, the P10K database contains 2929 genomes and transcriptomes, including 1096 newly sequenced datasets by P10K and 1833 publicly available datasets. It covers approximately 45% of the protist orders, with a particular emphasis on ciliates, which account for nearly a thousand genomes/transcriptomes and represent 53% coverage. Overall, the P10K database serves as an invaluable genetic resource repository for protist research and aims to expand further by incorporating additional sequenced data and advanced analysis tools, benefiting protist studies worldwide.

#### MPA

MPA (Mycobacteriaceae Phenome Atlas, https://www.biosino.org/mpa/) is a standardized atlas for the Mycobacteriaceae phenome based on heterogeneous sources. MPA includes a total of 82 microbial phenotypic traits of 10 755 strains from 236 species and 18 subspecies in Mycobacteriaceae. These traits were further classified into five categories and 20 subcategories of polyphasic phenotypes, as well as three categories and eight subcategories of functional phenotypes. The phenotypes were searchable and comparable from the website of MPA. The application of MPA may provide novel insights into the pathogenicity mechanism and antimicrobial targets of Mycobacteriaceae.

#### PPGR

PPGR (Perennial Plant Genomes and Regulation database, https://ngdc.cncb.ac.cn/ppgr/) serves as a public database dedicated to the exploration of perennial plant genomics and gene regulation (49). This resource encompasses data derived from 60 plant species, featuring richly annotated genomic information, 836 million protein-protein and transcription factor-target interactions, along with 8975 transcriptome samples representing environmental conditions and genetic backgrounds. The primary focus of PPGR centers on genes regulating critical processes in perennial plants, such as wood production, dormancy, terpene biosynthesis, and leaf senescence. Data sources comprise experiments, literature mining, public databases, and genomic predictions. With its user-friendly suite of multi-omics tools, PPGR will significantly contributes to the broader plant science community, extending its benefits far beyond the study of woody perennial plants.

## Recent updates

### Raw data & metadata

#### BioProject and BioSample

**Figure 3.**
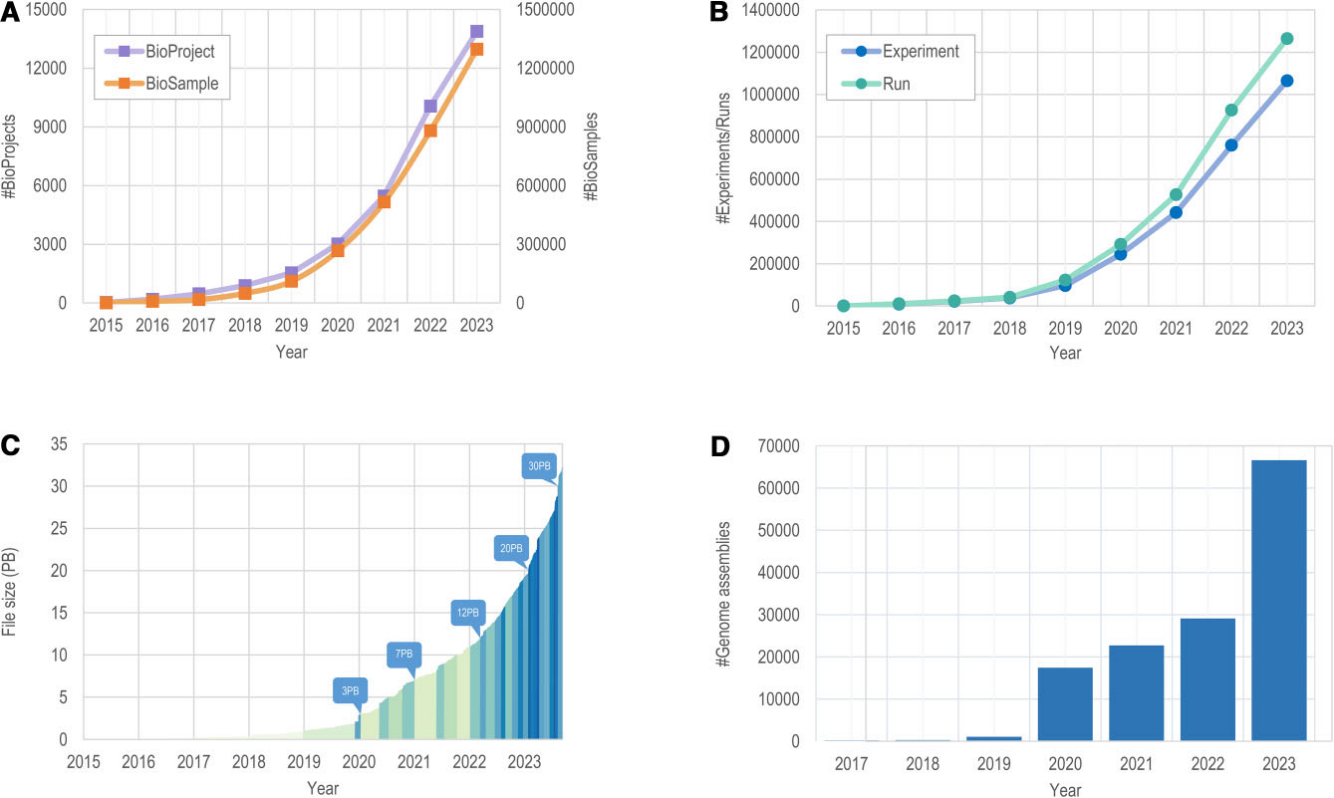
Statistics of data submissions to CNCB-NGDC. (**A**) Data statistics of BioProject and BioSample. (**B**) Data statistics of Experiments and Runs in GSA. (**C**) Timeline of data growth in GSA. (**D**) Statistics of genome assemblies in GWH. All statistics are regularly updated and publicly accessible at https://ngdc.cncb.ac.cn/bioproject, https://ngdc.cncb.ac.cn/biosample and https://ngdc.cncb.ac.cn/gsa and https://ngdc.cncb.ac.cn/gwh.

BioProject (https://ngdc.cncb.ac.cn/bioproject) and BioSample (https://ngdc.cncb.ac.cn/biosample) are two public repositories for biological research projects and samples, respectively. They gather descriptive metadata on biological projects and samples investigated in experiments and offer centralized access to all public projects and samples, along with cross-links to related data resources. As of August 2023, BioProject and BioSample have amassed a total of 13 487 biological projects and 1 244 954 biological samples submitted by 6438 users from 1549 organizations (Figure 3A). This represents a significant increase compared to the previous release in September, which had 7906 projects and 783 267 samples. Furthermore, this year, these two repositories have mirrored 709 261 projects and 34 622 211 samples from the INSDC data at NCBI.

#### GSA, GSA-Human and OMIX

The Genome Sequence Archive (GSA; https://ngdc.cncb.ac.cn/gsa) (50,51) is an archival database for raw sequence reads, which provides the global communities with free and open services for data submission, data storage and data sharing. GSA for Human (GSA-Human;https: //ngdc.cncb.ac.cn/gsa-human) (50), a sub-database of GSA, is a specialized data archive for human genetic omics data with controlled access and security services. As of August 2023, GSA and GSA-Human have collectively accumulated 1 032 023 experiments, 1 232 648 runs, and a total of 29.6 PB of data, demonstrating an exponential growth in data volumes (Figure 3B, C). In addition, GSA has integrated 25 695 978 experiments, 27 360 390 runs, and 4.5 PB of sequence files from the INSDC's data at NCBI SRA. The Open Archive for Miscellaneous Data database (OMIX; https://ngdc.cncb.ac.cn/omix) (50), as a member of the GSA family, strictly adheres to the FAIR principles and provides users with a platform to publish omics-based research outputs that are citable, shareable, and discoverable. As of August 2023, OMIX has archived 3384 submissions and 15 837 files with a size of 59.34 TB. Approximately 40% of the data files are related to human genetic resources, which are securely shared in a controlled access mode, requiring users to submit a simple application for access.

#### Database commons

Database Commons (https://ngdc.cncb.ac.cn/databasecommons) is a global catalog of biological databases that provides easy access and retrieval to a full collection of worldwide biological databases (52). It assesses the impact of databases and offers valuable statistics and trends. Currently, it catalogues a total of 6354 databases from around the world, encompassing 9808 publications and involving about 2100 organizations. This represents growth compared to the previous version in August 2022, which included 5831 databases and 8933 publications. Most databases have been curated by expert curators. In terms of database functionality updates, Database Commons started accepting open submissions of database from various institutions and universities around the world since the second half of 2022. The databases related to current research hotspots and frontiers are particularly curated. For example, a comprehensive collection of curated long non-coding RNA databases is compiled to facilitate an extensive review of this field (53). Furthermore, databases on SARS-CoV-2, rice, single cell, spatial omics, and immune research are newly curated. These databases can be easily accessed by clicking on the respective links located below the search box.

### Genome and variation

#### Genome warehouse

The Genome Warehouse (GWH; https://ngdc.cncb.ac.cn/gwh) is a valuable public resource for hosting genomic sequences, annotations, and metadata (54). By August 2023, the number of submitted genome assemblies has notably increased to 66 435, compared to 24 781 assemblies in September 2022 (Figure 3D). Among these, 19 350 genome assemblies from 1511 species have been released and published in 278 journal articles, indicating growth compared to 12 887 assemblies and 206 articles in September 2022. The recent data expansion in GWH is driven by Metagenome-Assembled Genomes (MAGs) and binned metagenomes. Notably, this update includes several enhancements such as the integration of 1 782 915 assemblies from INSDC, allowing for enhanced local searchability, browsability, and downloadability, along with detailed information pages for each assembly. Importantly, GWH is enhanced by incorporating a data request management system, which facilitates communication between data owners and applicants seeking controlled access data. Moreover, it is equipped with an advanced search system to enable categorical search and filtering, enhancing accessibility to both archived and integrated genome data. The continued expansion and improvements in GWH make it a valuable resource for advancing genomics research worldwide.

### Health and disease

#### RCoV19

The 2019 Novel Coronavirus Resource (RCoV19; https://ngdc.cncb.ac.cn/ncov) (55–58) is a comprehensive platform for the integration of SARS-CoV-2 genome data, variant monitoring, and risk pre-warning. As of August 2023, RCoV19 has integrated over 16.5 million SARS-CoV-2 sequences and metadata, among which ∼7.7 million have been further identified as complete and high-quality genome sequences for download analysis. Additionally, it has served over 3.5 million visitors from 182 countries/regions worldwide, with more than 17 billion data downloads in total. Over the past year, RCoV19 has undergone significant improvements in functionality. Firstly, it has implemented an advanced genome data curation model with an automated integration pipeline and optimized curation rules, enabling efficient daily data updates. Secondly, RCoV19 offers a global and regional lineage evolution monitoring platform and an outbreak risk pre-warning system, providing comprehensive insights into SARS-CoV-2 evolution and transmission patterns. Thirdly, a powerful interactive mutation spectrum comparison module allows users to analyze and compare mutation patterns, aiding in the detection of potential new lineages. Moreover, RCoV19 incorporates a comprehensive knowledgebase on mutation effects, serving as a valuable resource for retrieving information on the functional implications of specific mutations. In summary, RCoV19 is a crucial scientific resource that provides free, open access to valuable data, relevant information, and technical support in the global fight against COVID-19.

### Expression

#### Gene expression nebulas

Gene Expression Nebulas (GEN; https://ngdc.cncb.ac.cn/gen) is a data portal integrating transcriptomic profiles from both bulk and single-cell levels in various conditions across multiple species (59). The current version of GEN has undergone significant improvements and updates, particularly in ontology classification and data volume with 106 datasets and 5179 samples. GEN has systematically incorporated 34 gene expression profiling datasets related to 33 cancer types, encompassing 2768 samples. Furthermore, 30 rice-related datasets and 880 samples have been analyzed and included. Moreover, 42 gene expression profiling datasets (28 bulk and 16 scRNA-seq) and 1531 samples related to 10 new species derived from 33 original high-throughput sequencing projects have been added. Compared to the previous release in August 2022, the total number of incorporated datasets has increased from 469 to 575, covering 59 609 samples and 19 231 318 cells from 44 species, including 31 animals, 10 plants, 2 protists and 1 fungus. In terms of functionality, GEN has been improved by upgrading GENToolkit to facilitate prokaryotic transcriptome data with expression profiling and multiple downstream analysis in bulk RNA-seq level.

### Epigenomics

#### Editome disease knowledgebase

Editome Disease Knowledgebase (EDK, https://ngdc.cncb.ac.cn/edk) is a comprehensive database of editome-disease associations based on literature curation and integrative analysis (60). In its current version, EDK includes a total of 75 514 editing events, consisting of 826 experimentally validated endogenous and exogenous RNA editing events, as well as 74 688 abnormal editing events. These events span across 117 different diseases and are curated from 314 publications. Compared to the previous release in January 2019, the number of experimentally validated editing events has increased significantly from 248 to 826. Furthermore, by systematically integrating and analyzing 48 disease-associated RNA-seq datasets (comprising 2536 samples across 30 tissues) from GEN (59), the updated EDK encompasses a total of 577 341 new disease-associated editing sites, resulting in 18 690 508 abnormal RNA editing events that induce A-to-I and C-to-U RNA editing. In aspect of database functionality, EDK has been significantly upgraded with the addition of two user-friendly tools: Editing Identifier and Disease Predictor, with the aim to identify RNA editing events and provide a ranked list of editome-disease associations, respectively.

#### EWAS open platform

EWAS Open Platform (https://ngdc.cncb.ac.cn/ewas) incorporates data, knowledge, and toolkit for epigenome-wide association studies (EWAS) (61). Compared to the previous version in August 2022, the platform has undergone significant improvements. In terms of data, it has added 13 006 standardized and batch effect-corrected samples, covering 165 tissue types, 90 distinct diseases and 45 varied fields (62). In terms of knowledge, it includes 5203 new high-quality associations covering 47 traits through manual curation (63). Furthermore, EWAS Open Platform is functionally enhanced by developing an online analysis tool for batch effect correction and thus allowing users to integrate data directly from multiple sources (64). Users can obtain methylation levels after noise reduction by uploading original methylated and unmethylated signal value files or by entering the project ID in NCBI GEO. Currently, the platform encompasses standardized methylation array data from 146 678 samples across 265 fields, integrates 647 747 EWAS associations from 1043 published studies, and offers online tools for batch effect correction, enrichment analysis, annotation, and network visualization. Collectively, EWAS Open Platform aims to advance research into the roles of DNA methylation in development, aging, and diseases.

#### NucMap

NucMap (https://ngdc.cncb.ac.cn/nucmap) is a comprehensive database of genome-wide nucleosome positioning map across multiple species (65). The current version of NucMap includes 2718 nucleosome positioning information across 35 species, including animals, plants, fungi, and protozoa. In addition to nucleosome positioning data, NucMap integrates various other omics information such as mRNA expression, transcription factors (TFs), histones, and methylation data. Importantly, in the past year, the functionality of NucMap has been greatly improved from the following aspects. Firstly, NucMap newly facilitates the interpretation of gene regulation in humans by pre-analyzing and integrating 160 transcriptomes and 249 histone ChIP-seq data (including 31 types of histone modifications) specifically for human-related samples. Secondly, NucMap provides information of 180 102 474 potential TF binding sites across 27 species, allowing users to combine with collected ChIP-seq and RNA-seq data to infer the transcription process. Thirdly, a comparative analysis module is added to identify differential nucleosome regions, which can help users find potential regulatory regions. In summary, NucMap serves as a valuable resource for investigating the biological role of nucleosomes in genome regulation.

#### MethBank

The Methylation Bank (MethBank; https://ngdc.cncb.ac.cn/methbank) (66–68) is a comprehensive database of DNA methylation in multiple biological contexts across various species. Compared to last year, MethBank newly incorporates methylomes of two new model organisms of *Arabidopsis thaliana* and *Populus trichocarpa*, and expands methylation profiles in biological contexts, especially in terms of disease, environment, and development. Currently, MethBank systematically incorporates whole-genome single-base resolution methylomes of 2101 high-quality samples from 241 projects in 25 species, representing a 45% increase over the previous release (1449 samples from 199 projects in 23 species). To characterize DNA methylation signatures in more biological contexts, 168 416 058 methylation profiles of genes, 4 961 814 methylated CpG islands, and 60 105 424 differentially methylated regions are newly provided based on these sequencing data. In addition to the enrichment of data volume, MethBank is also significantly upgraded by integrating more featured DMGs associated with biological contexts, growing from 2124 entries to 2905 entries curated from 278 publications across 147 tissues/cell lines, 151 diseases, and 12 biological contexts. To further improve the usability of the DMR toolkit, MethBank has been updated by integrating more species and optimizing enrichment analysis.

### Biodiversity

#### TCOD

The Tropical Crop Omics Database (TCOD, https://ngdc.cncb.ac.cn/tcod) is a comprehensive multi-omics platform dedicated to tropical crop research (69). The latest version of TCOD brings substantial enhancements in data volume, gene function annotation and analysis tools. Currently, TCOD contains 34 chromosome-level *de novo* assemblies, 1 255 004 genes, 282 436 992 unique variants, 88 transcriptomic profiles, and 13 381 germplasm items in 15 representative species, compared to 14 chromosome-level genome assemblies, 565 185 genes, 111 934 324 unique variants and 10 433 germplasm items in five tropical crops in the previous version (September 2022). Furthermore, TCOD improves its functionality by utilizing multiple databases for consistent gene functional annotation and furnishing gene homology relationships across species. In addition to the enhancement of existing tools, a series of new tools such as Primer Design, GO Enrichment, KEGG Enrichment, Synteny Viewer, and Homolog Finder have been developed and deployed in TCOD.

### Tools

#### BIG Search

BIG Search (https://ngdc.cncb.ac.cn/search) is a distributed and scalable full-text search engine for a large number of biological resources and provides one-stop cross-database search services for the global research community. In its current version, BIG Search integrates both the NGDC internal databases and 55 partner databases (https://ngdc.cncb.ac.cn/partners), resulting in a total of 1.472 billion data entries and over 1.4 terabytes of data. Additionally, it incorporates 35 important NCBI biological databases (70) and 165 biological datasets from EBI (71) through API. BIG Search offers advanced search functions and cross-database search services for numerous data resources, providing users with a more convenient and efficient means of retrieving data.

## Concluding remarks

With the exponential growth of multi-omics data, CNCB-NGDC is committed to continuously providing a comprehensive suite of newly developed and updated database resources, aiming to facilitate data submissions and offer value-added annotations and curated knowledge for the global research community. CNCB-NGDC is actively engaged in various ongoing efforts, including but not limited to, automating data submission processes, curating data, integrating and analyzing data, upgrading infrastructure for efficient storage and transmission of big data, and developing new tools and pipelines for multi-omics data deep mining. These endeavors are aimed at supporting the analysis and interpretation of big data in a more streamlined and efficient manner. As one of the major global centers in genomics and bioinformatics, CNCB-NGDC is dedicated to expanding its resources and services to provide a comprehensive range of data resources and services that support knowledge discovery for a wide array of research activities in the fields of life and health sciences.

## Data Availability

All resources and services are publicly available in the home page of CNCB-NGDC (https://ngdc.cncb.ac.cn).

## Notes

Full list provided in Appendix.

## Appendix

**Corresponding author:** Yiming Bao^1,2,3,*^

**Co-corresponding authors:** Zhang Zhang^1,2,3,*^, Wenming Zhao^1,2,3,*^, Jingfa Xiao^1,2,3,*^, Shunmin He^4,*^, Guoqing Zhang^5,*^, Yixue Li^5,6,*^, Guoping Zhao^5,7,*^, Runsheng Chen^4,*^

**CNCB-NGDC MEMBERS (Arranged by project role and then by contribution except for Team Leader (TL), as indicated**)

**GenBase:** Congfan Bu^1,2,#^, Xinchang Zheng^1,2,#^, Xuetong Zhao^1,2,#^, Tianyi Xu^1,2,#^, Xue Bai^1,2,#^, Yaokai Jia^1,2^, Meili Chen^1,2^, Lili Hao^1,2,3^, Jingfa Xiao^1,2,3^, Zhang Zhang^1,2,3^, Wenming Zhao^1,2,3^, Bixia Tang^1,2,#^, Yiming Bao^1,2,3,*^

**OBIA:** Enhui Jin^1,2,3,#^, Dongli Zhao^8,#^, Gangao Wu^1,2,3,#^, Junwei Zhu^1,2^, Zhonghuang Wang^1,2,3^, Zhiyao Wei^8^, Sisi Zhang^1,2^, Anke Wang^1,2^, Bixia Tang^1,2^, Xu Chen^1,2^, Yanling Sun^1,2,#^, Zhe Zhang^9,#^, Wenming Zhao^1,2,3,*^, Yuanguang Meng^8,9,#^

**OPIA:** Yongrong Cao^1,2,3,#^, Dongmei Tian^1,2,#^, Zhixin Tang^3,10^, Xiaonan Liu^1,2,3^, Weijuan Hu^10,#^, Zhang Zhang^1,2,3,*^, Shuhui Song^1,2,3,#^

**CROST:** Guoliang Wang^1,2,3,#^, Song Wu^1,2,3,#^, Zhuang Xiong^11,#^, Hongzhu Qu^1,2,3,#^ (TL), Xiangdong Fang^1,2,3,#^ (TL), Yiming Bao^1,2,3,*^ (TL)

**SMDB:** Ruifang Cao^5,#^, Yunchao Ling^5,#^, Jiayue Meng^5,#^, Qinwen He^5^, Yixue Li^5,6,*^, Guoqing Zhang^5,*^

**HervD Atlas:** Cuidan Li^12,#^, Qiheng Qian^1,2,3,#^, Chenghao Yan^12,3,#^, Mingming Lu^1,2,#^, Pan Li^12,3^, Zhuojing Fan^1,2^, Wenyan Lei^12,3^, Kang Shang^12,3^, Peihan Wang^12,3^, Jie Wang^12,3^, Tianyi Lu^12,3^, Yuting Huang^13^, Hongwei Yang^13^, Haobin Wei^12,3^, Jingfa Xiao^1,2,3,*^ (TL), Fei Chen^3,12,14,#^ (TL)

**HALL:** Hao Li^3,15,#^, Song Wu^1,2,3,#^ , Jiaming Li^3,15,#^, Zhuang Xiong^11,#^, Kuan Yang^3,15,16,#^, Weidong Ye^17,#^, Jie Ren^3,15,16,18,#,^Yun-Gui Yang^15,19,#^, Feng Zhang^17,#^, Guang-Hui Liu^3,18,20,21,22,23,24,#^, Yiming Bao^1,2,3,*^, Weiqi Zhang^3,15,18,24,#^

**MACdb:** Yanling Sun^1,2,#^, Xinchang Zheng^1,2,#^, Guoliang Wang^1,2,3,#^, Yibo Wang^1,2,3,#^, Xiaoning Chen^1,2,3^, Jiani Sun^1,2,3,16^, Zhuang Xiong^1,2,3^, Sisi Zhang^1,2^, Zhuojing Fan^1,2^, Congfan Bu^1,2^, Yiming Bao^1,2,3,*^, Wenming Zhao^1,2,3,*^

**NAFLDkb:** Tingjun Xu^25,26,#^, Wenxing Gao^25,#^, Lixin Zhu^27,28,#^, Guoqing Zhang^5,*^, Ruixin Zhu^25,#^, Dingfeng Wu^29,#^

**BioKA:** Yibo Wang^1,2,3,#^, Yihao Lin^1,2,3,#^, Sicheng Wu^1,2,3,#^, Jiani Sun^1,2,3^, Yuyan Meng^1,2,3^, Enhui Jin^1,2,3^, Demian Kong^1,2,3^, Guangya Duan^1,2,3^, Shaoqi Bei^30^, Zhuojing Fan^1,2^, Gangao Wu^1^, Lili Hao^1,2,3^, Shuhui Song^1,2,3^, Bixia Tang^1,2,#^, Wenming Zhao^1,2,3,*^

**RePoS:** Huaxia Luo^4,#^, Peng Zhang^4,#^, Wanyu Zhang^4^, Yu Zheng^4^, Di Hao^4^, Yirong Shi^4^, Yiwei Niu^4^, Tingrui Song^4^, Yanyan Li^4^

**TargetGene:** Shiqi Lin^3,15,#^, Song Wu^1,2,3,#^, Wei Zhao^1,2,3^, Zhanjie Fang^3,15^, Hongen Kang^3,15^, Xinxuan Liu^3,15^, Siyu Pan^3,15^, Fudong Yu^31,#^, Yiming Bao^1,2,3,*^, Peilin Jia^3,15,#^ (TL)

**PGG.SV:** Yimin Wang^5,#^, Yunchao Ling^5,#^, Jiao Gong^32,33,#^, Shaohua Fan^32,#^, Guoqing Zhang^5,*^, Shuhua Xu^32,33,#^

**PlantPan:** Meiye Jiang^1,2,3,#^, Qiheng Qian^1,2,3,#^, Jingyao Zeng^1,2,^, Meili Chen^1,2,^, Jingfa Xiao^1,2,3,*^

**NTM-DB:** Tianyi Lu^3,12,#^, Cuidan Li^3,#^, Haobin Wei^3,12,16,#^, Yadong Zhang^1,2,#^, Zhuojing Fan^1,2^, Xiaoyuan Jiang^3^, Jie Wang^3,12^, Peihan Wang^3,12^, Yuting Huang^13^, Hongwei Yang^13^, Jingfa Xiao^1,2,3,*^ (TL), Fei Chen^3,12,14,#^ (TL)

**SoyOmics:** Yucheng Liu^10,#^, Yang Zhang^1,2,3,#^, Xiaonan Liu^1,2,3,#^, Yanting Shen^10,#^, Dongmei Tian^1,2^, Xiaoyue Yang^10^, Shulin Liu^10^, Lingbin Ni^3,10^, Zhang Zhang^1,2,3,*^, Shuhui Song^1,2,3,#^, Zhixi Tian^3,10,#^

**The P10K Database:** Xinxin Gao^3,34,#^, Kai Chen^34,#^, Jie Xiong^34,35,#^, Dong Zou^1,2,#^, Fangdian Yang^34^, Yingke Ma^1,2^, Chuanqi Jiang^34^, Xiaoxuan Gao^36^, Guangying Wang^34^, Siyu Gu^3,34^, Peng Zhang^34,^, Shuai Luo^34^, Kaiyao Huang^34,37^, Yiming Bao^1,2,3^, Zhang Zhang^1,2,3,*^, Lina Ma^1,2,3,#^ (TL), Wei Miao^34,37,38,#^ (TL)

**MPA:** Wan Liu^5,#^, Hui Cen^5,#^, Zhile Wu^5,39,#^, Haokui Zhou^40^, Shuo Chen^40^, Xilan Yang^40^, Guoping Zhao^5,7,*^, Guoqing Zhang^5,*^

**PPGR:** Sen Yang^41,42,43,#^, Wenting Zong^1,2,3,#^, Yiming Bao^1,2,3,*^, Rujiao Li^1,2,3,#^ (TL), Jianbo Xie^41,42,43,#^

**BioProject & BioSample & GSA & GSA-Human & BIG Submission:** Xu Chen^1,2,#^, Tingting Chen^1,2,#^, Lili Dong^1,2,#^, Yanling Sun^1,2,#^, Sisi Zhang^1,2,#^, Caixia Yu^1,2^, Bixia Tang^1,2^, Junwei Zhu^1,2^, Yubo Zhou^1,2^, Zhuojing Fan^1,2^, Shuang Zhai^1,2^, Yubin Sun^1,2^, Qiancheng Chen^1,2^, Xiaoyu Yang^1,2^, Xin Zhang^1,2^, Zhengqi Sang^1,2^, Yonggang Wang^1,2^, Yilin Zhao^1,2^, Huanxin Chen^1,2^, Li Lan^1,2^, Yanqing Wang^1,2,#^ (TL), Wenming Zhao^1,2,3,*^ (TL)

**OMIX:** Anke Wang^1,2,#^, Caixia Yu^1,2,#^, Sisi Zhang^1,2,#^ (TL)

**Database Commons:** Yuxin Qin^1,2,3,#^, Xinyu Zhou^1,2,3,44,#^, Yue Qi^1,2,3,#^, Yuanyuan Cheng^1,2,3,16,#^, Nan Yang^1,2,3,44^, Dong Zou^1,2^, Lin Liu^1,2^, Lina Ma^1,2,3,#^ (TL)

**Genome Warehouse:** Yingke Ma^1,2,#^, Yaokai Jia^1,2,#^, Xuetong Zhao^1,2,#^, Meili Chen^1,2,#^ (TL)

**RCoV19:** Cuiping Li^1,2,#^, Lina Ma^1,2,3,#^, Dong Zou^1,2,#^, Rongqin Zhang^1,2,3,16,#^, Xue Bai^1,2^, Lun Li^1,2^, Junwei Zhu^1,2^, Wei Zhao^1,2,3^, Gangao Wu^1,2,3^, Tianhao Huang^1,2,3^, Enhui Jin^1,2,3^, Hailong Kang^1,2,3^, Zhang Zhang^1,2,3^, Wenming Zhao^1,2,3^, Yongbiao Xue^1,2,3^, Yiming Bao^1,2,3,*^ (TL), Shuhui Song^1,2,3,#^ (TL)

**GEN:** Tianyi Xu^1,2,#^, Ming Chen^1,2,3,#^, Tongtong Zhu^1,2,3,#^, Rong Pan^1,2,3,#^, Dong Zou^1,2,#^, Yuanyuan Cheng^1,2,3,#^, Yuan Chu^1,2,3,#^, Guangyi Niu^1,2^, Lili Hao^1,2,3,#^ (TL), Zhang Zhang^1,2,3,*^

**EDK:** Tongtong Zhu^1,2,3,#^, Dong Zou^1,2,#^, Guangyi Niu^1,2,#^, Tianyi Xu^1,2,#^, Yuan Chu^1,2,3,#^, Yuansheng Zhang^1,2,3^, Ming Chen^1,2,3^, Rong Pan^1,2,3^, Yuanyuan Cheng^1,2,3^, Zhao Li^1,2,3^, Shuai Jiang^1,2^, Lili Hao^1,2,3,#^, Zhang Zhang^1,2,3,*^

**EWAS Open Platform:** Fei Yang^1,2,#^, Zhuang Xiong^11,#^, Song Wu^1,2,3,#^ , Wenting Zong^1,2,3^, Rujiao Li^1,2,3,#^ (TL)

**NucMap:** Zhi Nie^1,2,3,#^, Shuhuan Yu^1,2,3,#^, Yongbing Zhao^45,#^, Jialin Mai^1,2,3^, Hao Gao^1,2,3^, Zhuojing Fan^1,2^, Yiming Bao^1,2,3,*^, Rujiao Li^1,2,3,#^ (TL), Jingfa Xiao^1,2,3,*^

**MethBank:** Mochen Zhang^1,2,3,#^, Fei Yang^1,2,#^, Wenting Zong^1,2,3,#^, Yiran Zhang^1,2,3,#^, Dong Zou^1,2,#^, Yiyun Liu^1,2,3^, Xutong Guo^1,2,3^, Rujiao Li^1,2,#^ (TL)

**TCOD:** Hailong Kang^1,2,3,#^, Tianhao Huang^1,2,3,#^, Guangya Duan^1,2,3,#^, Yuyan Meng^1,2,3^, Xiaoning Chen^1,2,3^, Shuang He^46^, Zhiqiang Xia^46^, Xincheng Zhou^47^, Jinquan Chao^48^, Bixia Tang^1,2^, Zhonghuang Wang^1,2,3^, Junwei Zhu^1,2^, Zhenglin Du^1,2^, Yanlin Sun^1,2^, Sisi Zhang^1,2^, Jingfa Xiao^1,2,3^, Weimin Tian^48^, Wenquan Wang^46,#^, Wenming Zhao^1,2,3,*^

**BIG Search:** Dong Zou^1,2^

**Writing Group:** Shuai Jiang^1,2,#^, Zhuojing Fan^1,2^, Wenming Zhao^1,2,3,*^, Jingfa Xiao^1,2,3,*^ , Zhang Zhang^1,2,3,*^, Yiming Bao^1,2,3,*^

**CNCB-NGDC PARTNERS (Listed in alphabetical order by database names**)

**Animal-APA:** Weiwei Jin^49^, Jing Gong^49^

**Animal-eRNA:** Weiwei Jin^49^, Jing Gong^49^

**Animal-SNPAtlas:** Xiaohui Niu^49^, Jing Gong^49^

**AnimalTFDB:** Wen-Kang Shen^50^, An-Yuan Guo^50^

**BBCancer:** Zhixiang Zuo^51^, Jian Ren^51^

**CancerSEA:** Xinxin Zhang^52^, Yun Xiao^52^, Xia Li^52^

**CellMarker:** Xinxin Zhang^52^, Yun Xiao^52^, Xia Li^52^

**CGDB:** Dan Liu^50^, Chi Zhang^50^, Yu Xue^50^

**CGGA:** Zheng Zhao^53^, Tao Jiang^53^

**circAtlas:** Wanying Wu^54^, Fangqing Zhao^54^

**CirFunBase:** Xianwen Meng^55^, Ming Chen^55^

**CPLM:** Yujie Gou^50^, Miaomiao Chen^50^, Yu Xue^50^

**dbPSP & THANATOS:** Di Peng^50^, Yu Xue^50^

**DEG & DoriC:** Hao Luo^56,57,58^, Feng Gao^56,57,58^

**DrLLPS:** Danyang Xu^50^, Jianzhen Peng^50^, Yu Xue^50^

**eLMSG:** Wan Liu^5^, Yunchao Ling^5^, Ruifang Cao^5^, Guoqing Zhang^5^

**EPSD & WERAM:** Yuxiang Wei^50^, Leming Xiao^50^, Yu Xue^50^

**EVAtlas:** Chun-Jie Liu^50^, An-Yuan Guo^50^

**EVmiRNA:** Gui-Yan Xie^50^, An-Yuan Guo^50^

**GenTree:** Hao Yuan^3,59^, Tianhan Su^3,59^, Yong E. Zhang^3,59,60^

**GTDB:** Chenfen Zhou^5^, Pengyu Wang^5^, Guoqing Zhang^5^

**HCL:** Yincong Zhou^55^, Ming Chen^55^, Guoji Guo^61^

**hTFtarget:** Qiong Zhang^50^, An-Yuan Guo^50^

**iEKPD:** Shanshan Fu^50^, Miaoying Zhao^50^, Yu Xue^50^

**iPCD:** Dachao Tang^50^, Yu Xue^50^

**iUUCD:** Weizhi Zhang^50^, Yu Xue^50^

**LeukemiaDB:** Mei Luo^50^, An-Yuan Guo^50^

**lnCAR:** Yubin Xie^51^, Jian Ren^51^

**lncRNASNP2:** Ya-Ru Miao^50^, An-Yuan Guo^50^

**lncRNASNP v3:** An-Yuan Guo^50^, Jing Gong^49^

**MCA:** Yincong Zhou^55^, Ming Chen^55^, Guoji Guo^61^

**MiCroKiTS:** Xinhe Huang^50^, Zihao Feng^50^, Yu Xue^50^

**miRNASNP:** Chun-Jie Liu^50^, An-Yuan Guo^50^

**msRepDB:** Xingyu Liao^62,63^, Xin Gao^62^, Jianxin Wang^63^

**ncRNA-eQTL:** Jiang Li^49^, Jing Gong^49^

**Pancan-mnvQTL:** Xiaohui Niu^49^, Jing Gong^49^

**PEA:** Guiyan Xie^50^, An-Yuan Guo^50^

**PceRBase:** Chunhui Yuan^55^, Ming Chen^55^

**PlantRegMap:** Dechang Yang^64^, Feng Tian^64^, Ge Gao^64^

**Plant-ImputeDB:** Xiaohui Niu^49^, Qing-Yong Yang^49^, Jing Gong^49^

**PncStres:** Wenyi Wu^55^, Ming Chen^55^

**PTMD:** Cheng Han^50^, Yu Xue^50^, Qinghua Cui^65,66^

**RhesusBase:** Juntian Qi^67^, Chuan-Yun Li^67^

**RMVar:** XiaoTong Luo^51^, Jian Ren^51^

**SEECancer:** Xinxin Zhang^52^, Yun Xiao^52^, Xia Li^52^

**SEGreg:** Qing Tang^50^, An-Yuan Guo^50^

**SNP2APA:** An-Yuan Guo^50^, Jing Gong^49^

**VFDB:** Bo Liu^68^, Jian Yang^68^

**ZCURVE_CoVdb:** Hao Luo^56,57,58^, Feng Gao^56,57,58^

*To whom correspondence should be addressed: Yiming Bao (baoym@big.ac.cn).

Correspondence may also be addressed to Zhang Zhang (zhangzhang@big.ac.cn), Wenming Zhao (zhaowm@big.ac.cn), Jingfa Xiao (xiaojingfa@big.ac.cn), Shunmin He (heshunmin@ibp.ac.cn), Guoqing Zhang (gqzhang@picb.ac.cn), Yixue Li (yxli@sibs.ac.cn), Guoping Zhao (gpzhao@sibs.ac.cn) and Runsheng Chen (crs@ibp.ac.cn).

^#^The authors wish it to be known that, in their opinion, these authors should be regarded as Joint First Authors.

^1^National Genomics Data Center & CAS Key Laboratory of Genome Sciences and Information, Beijing Institute of Genomics, Chinese Academy of Sciences, Beijing 100101, China

^2^China National Center for Bioinformation, Beijing 100101, China

^3^University of Chinese Academy of Sciences, Beijing 100049, China

^4^Key Laboratory of Epigenetic Regulation and Intervention, Institute of Biophysics, Chinese Academy of Sciences, Beijing 100101, China

^5^National Genomics Data Center & Bio-Med Big Data Center, CAS Key Laboratory of Computational Biology, Shanghai Institute of Nutrition and Health, University of Chinese Academy of Sciences, Chinese Academy of Science, Shanghai 200031, China

^6^Guangzhou Laboratory, Guangzhou 510005, China

^7^Hangzhou Institute for Advanced Study, University of Chinese Academy of Sciences, Hangzhou 310024, China

^8^Chinese People's Liberation Army (PLA) Medical School, Beijing 100853, China

^9^Department of Obstetrics and Gynecology, Seventh Medical Center of Chinese PLA General Hospital, Beijing 100700, China

^10^State Key Laboratory of Plant Cell and Chromosome Engineering, Institute of Genetics and Developmental Biology, Chinese Academy of Sciences, Beijing 100101, China

^11^Interdisciplinary Institute for Medical Engineering, Fuzhou University, Fuzhou 350002, China

^12^CAS Key Laboratory of Genome Sciences and Information, Beijing Institute of Genomics, Chinese Academy of Sciences and China National Center for Bioinformation, Beijing 100101, China

^13^State Key Laboratory of Elemento-Organic Chemistry, College of Chemistry, Nankai University, Tianjin 300071, China

^14^Beijing Key Laboratory of Genome and Precision Medicine Technologies, Beijing 100101, China

^15^CAS Key Laboratory of Genomic and Precision Medicine, Beijing Institute of Genomics, Chinese Academy of Sciences and China National Center for Bioinformation, Beijing 100101, China

^16^Sino-Danish College, University of Chinese Academy of Sciences, Beijing 100049, China

^17^The Joint Innovation Center for Engineering in Medicine, Quzhou People's Hospital, Quzhou 324000, China

^18^Institute for Stem cell and Regeneration, CAS, Beijing 100101, China

^19^State Key Laboratory of Stem Cell and Reproductive Biology, Institute of Zoology, Chinese Academy of Sciences, Beijing, 100101, China

^20^State Key Laboratory of Membrane Biology, Institute of Zoology, Chinese Academy of Sciences, Beijing 100101, China

^21^Beijing Institute for Stem Cell and Regenerative Medicine, Beijing 100101, China

^22^Advanced Innovation Center for Human Brain Protection, and National Clinical Research Center for Geriatric Disorders, Xuanwu Hospital Capital Medical University, Beijing 100053, China

^23^Aging Translational Medicine Center, Xuanwu Hospital, Capital Medical University, Beijing 100053, China

^24^Aging Biomarker Consortium, Beijing, 100101, China

^25^Putuo People's Hospital, School of Life Sciences and Technology, Tongji University, Shanghai 200060, China

^26^Shanghai Institute of Organic Chemistry, Chinese Academy of Sciences, ^345^ LingLing Road, Shanghai 200032, China

^27^Guangdong Institute of Gastroenterology; Guangdong Provincial Key Laboratory of Colorectal and Pelvic Floor Diseases; Biomedical Innovation Center, Sun Yat-sen University, Guangzhou 510655, China

^28^Department of General Surgery, The Sixth Affiliated Hospital of Sun Yat-sen University, Guangzhou 510655, China

^29^National Clinical Research Center for Child Health, the Children's Hospital, Zhejiang University School of Medicine, Hangzhou 310058, Zhejiang, China

^30^Qilu University of Technology (Shandong Academy of Sciences), Beijing 250353, China

^31^Shanghai-MOST Key Laboratory of Health and Disease Genomics, NHC Key Lab of Reproduction Regulation, Shanghai Institute for Biomedical and Pharmaceutical Technologies, Shanghai, China, 200237

^32^State Key Laboratory of Genetic Engineering, Center for Evolutionary Biology, Collaborative Innovation Center of Genetics and Development, School of Life Sciences, Fudan University, Shanghai 200438, China

^33^Human Phenome Institute, Zhangjiang Fudan International Innovation Center, and Ministry of Education Key Laboratory of Contemporary Anthropology, Fudan University, Shanghai 201203, China

^34^Institute of Hydrobiology, Chinese Academy of Sciences, Wuhan 430072, China

^35^Key Laboratory of Breeding Biotechnology and Sustainable Aquaculture, Chinese Academy of Sciences, Wuhan 430072, China

^36^Shandong University of Technology, Zibo255000, China

^37^Key laboratory of Lake and Watershed Science for Water Security, Chinese Academy of Sciences, Nanjing 210008, China

^38^Hubei Hongshan Laboratory, Wuhan 430070, China

^39^Shanghai Southgene Technology Co., Ltd., Shanghai 201210, China

^40^Institute of Synthetic Biology, Shenzhen Institutes of Advanced Technology, Chinese Academy of Sciences, Shenzhen 518055, China

^41^State Key Laboratory of Tree Genetics and Breeding, College of Biological Sciences and Technology, Beijing Forestry University, Beijing 100083, China

^42^National Engineering Research Center of Tree Breeding and Ecological Restoration, College of Biological Sciences and Technology, Beijing Forestry University, Beijing 100083, China

^43^The Tree and Ornamental Plant Breeding and Biotechnology Laboratory of National Forestry and Grassland Administration, Beijing Forestry University, Beijing 100083, China

^44^School of Future Technology, University of Chinese Academy of Sciences, Beijing 100049, China

^45^Center for Cell Lineage and Development, CAS Key Laboratory of Regenerative Biology, Guangzhou Institutes of Biomedicine and Health, Chinese Academy of Sciences, Guangzhou 510530, China

^46^Sanya Nanfan Research Institute, Hainan University, Sanya 572025, China

^47^Institute of Tropical Biosciences and Biotechnology, Chinese Academy of Tropical Agricultural Sciences, Haikou 571101, China

^48^Rubber Research Institute, Chinese Academy of Tropical Agricultural Sciences, Haikou 571101, China

^49^Hubei Key Laboratory of Agricultural Bioinformatics, College of Informatics, Huazhong Agricultural University, Wuhan 430070, P.R. China

^50^Department of Thoracic Surgery, West China Biomedical Big Data Center, West China Hospital, Sichuan University, Chengdu 610041, China

^51^State Key Laboratory of Oncology in South China, Cancer Center, Collaborative Innovation Center for Cancer Medicine, School of Life Sciences, Sun Yat-sen University, Guangzhou 510060, China

^52^College of Bioinformatics Science and Technology, Harbin Medical University, Harbin, Heilongjiang 150081, China

^53^Beijing Neurosurgical Institute, Capital Medical University, Beijing 100070, China

^54^Beijing Institutes of Life Science, Chinese Academy of Sciences, Beijing 100101, China

^55^Department of Bioinformatics, College of Life Sciences; The First Affiliated Hospital, School of Medicine, Zhejiang University, Hangzhou 310058, China

^56^Department of Physics, School of Science, Tianjin University, Tianjin 300072, China

^57^Frontiers Science Center for Synthetic Biology and Key Laboratory of Systems Bioengineering (Ministry of Education), Tianjin University, Tianjin 300072, China

^58^SynBio Research Platform, Collaborative Innovation Center of Chemical Science and Engineering (Tianjin), Tianjin 300072, China

^59^Key Laboratory of Zoological Systematics and Evolution and State Key Laboratory of Integrated Management of Pest Insects and Rodents, Institute of Zoology, Chinese Academy of Sciences, Beijing 100101, China

^60^CAS Center for Excellence in Animal Evolution and Genetics, Chinese Academy of Sciences, Kunming, Yunnan 650223, China

^61^Center for Stem Cell and Regenerative Medicine, Zhejiang University School of Medicine, Hangzhou, China

^62^Computational Bioscience Research Center (CBRC), Computer, Electrical and Mathematical Sciences and Engineering Division, King Abdullah University of Science and Technology (KAUST), Thuwal 23955, Saudi Arabia

^63^Hunan Provincial Key Lab on Bioinformatics, School of Computer Science and Engineering, Central South University, Changsha 410083, China

^64^State Key Laboratory of Protein and Plant Gene Research, School of Life Sciences, Biomedical Pioneering Innovative Center (BIOPIC) & Beijing Advanced Innovation Center for Genomics (ICG), Center for Bioinformatics (CBI), Peking University, Beijing 100871, China

^65^Department of Biomedical Informatics, School of Basic Medical Sciences, MOE Key Lab of Cardiovascular Sciences, Center for Noncoding RNA Medicine, Peking University, Beijing 100190, China

^66^Center of Bioinformatics, Key Laboratory for NeuroInformation of Ministry of Education, School of Life Science and Technology, University of Electronic Science and Technology of China, Chengdu, Sichuan 610054, China

^67^Beijing Key Laboratory of Cardiometabolic Molecular Medicine, Institute of Molecular Medicine, College of Future Technology, Peking University, Beijing, China

^68^NHC Key Laboratory of Systems Biology of Pathogens, Institute of Pathogen Biology, Chinese Academy of Medical Sciences & Peking Union Medical College, Beijing, China

## References

[1] Bao Y. , XueY. From BIG Data Center to China National Center for Bioinformation. Genomics Proteomics Bioinformatics. 2023; 10.1016/j.gpb.2023.10.001.37832784

[2] Genomes Project, C. Auton A. , Brooks L.D., Durbin R.M., Garrison E.P., Kang H.M., Korbel J.O., Marchini J.L., McCarthy S., McVean G.A. et al. A global reference for human genetic variation. Nature. 2015; 526:68–74. 26432245 10.1038/nature15393 PMC4750478

[3] Cancer Genome Atlas Research, N. Weinstein J.N. , Collisson E.A., Mills G.B., Shaw K.R., Ozenberger B.A., Ellrott K., Shmulevich I., Sander C., Stuart J.M. The cancer genome atlas pan-cancer analysis project. Nat. Genet. 2013; 45:1113–1120. 24071849 10.1038/ng.2764 PMC3919969

[4] Bycroft C. , Freeman C., Petkova D., Band G., Elliott L.T., Sharp K., Motyer A., Vukcevic D., Delaneau O., O’Connell J. et al. The UK Biobank resource with deep phenotyping and genomic data. Nature. 2018; 562:203–209. 30305743 10.1038/s41586-018-0579-z PMC6786975

[5] Choi Y.H. , Kim J.K. Dissecting cellular heterogeneity using single-cell RNA sequencing. Mol. Cells. 2019; 42:189–199. 30764602 10.14348/molcells.2019.2446 PMC6449718

[6] Griffiths J.A. , Scialdone A., Marioni J.C. Using single-cell genomics to understand developmental processes and cell fate decisions. Mol. Syst. Biol. 2018; 14:e8046. 29661792 10.15252/msb.20178046 PMC5900446

[7] Cheng S. , Li Z., Gao R., Xing B., Gao Y., Yang Y., Qin S., Zhang L., Ouyang H., Du P. et al. A pan-cancer single-cell transcriptional atlas of tumor infiltrating myeloid cells. Cell. 2021; 184:792–809. 33545035 10.1016/j.cell.2021.01.010

[8] Jovic D. , Liang X., Zeng H., Lin L., Xu F., Luo Y. Single-cell RNA sequencing technologies and applications: a brief overview. Clin. Transl. Med. 2022; 12:e694. 35352511 10.1002/ctm2.694 PMC8964935

[9] Chen L. , Fan R., Tang F. Advanced single-cell Omics Technologies and Informatics tools for genomics, proteomics, and bioinformatics analysis. Genomics Proteomics Bioinformatics. 2021; 19:343–345. 34923125 10.1016/j.gpb.2021.12.001 PMC8864189

[10] Wang R. , Peng G., Tam P.P.L., Jing N. Integration of computational analysis and spatial transcriptomics in single-cell studies. Genomics Proteomics Bioinformatics. 2023; 21:13–23. 35901961 10.1016/j.gpb.2022.06.006 PMC10372908

[11] CNCB-NGDC Members and Partners Database resources of the National Genomics Data Center, China National Center for Bioinformation in 2023. Nucleic Acids Res. 2023; 51:D18–D28. 36420893 10.1093/nar/gkac1073 PMC9825504

[12] CNCB-NGDC Members and Partners Database Resources of the National Genomics Data Center, China National Center for Bioinformation in 2022. Nucleic Acids Res. 2022; 50:D27–D38. 34718731 10.1093/nar/gkab951 PMC8728233

[13] CNCB-NGDC Members and Partners Database Resources of the National Genomics Data Center, China National Center for Bioinformation in 2021. Nucleic Acids Res. 2021; 49:D18–D28. 36420893 10.1093/nar/gkac1073 PMC9825504

[14] National Genomics Data Center Members and Partners Database resources of the National Genomics Data Center in 2020. Nucleic Acids Res. 2020; 48:D24–D33. 31702008 10.1093/nar/gkz913 PMC7145560

[15] BIG Data Center Members Database resources of the BIG Data Center in 2019. Nucleic Acids Res. 2019; 47:D8–D14. 30365034 10.1093/nar/gky993 PMC6323991

[16] BIG Data Center Members Database resources of the BIG Data Center in 2018. Nucleic Acids Res. 2018; 46:D14–D20. 29036542 10.1093/nar/gkx897 PMC5753194

[17] BIG Data Center Members The BIG Data Center: from deposition to integration to translation. Nucleic Acids Res. 2017; 45:D18–D24. 27899658 10.1093/nar/gkw1060 PMC5210546

[18] Jiang S. , Du Q., Feng C., Ma L., Zhang Z. CompoDynamics: a comprehensive database for characterizing sequence composition dynamics. Nucleic Acids Res. 2022; 50:D962–D969. 34718745 10.1093/nar/gkab979 PMC8728180

[19] Wang Y.Y. , Kang H., Xu T., Hao L., Bao Y., Jia P. CeDR Atlas: a knowledgebase of cellular drug response. Nucleic Acids Res. 2022; 50:D1164–D1171. 34634794 10.1093/nar/gkab897 PMC8728137

[20] Cao J. , Zhang Y., Tan S., Yang Q., Wang H.-L., Xia X., Luo J., Guo H., Zhang Z., Li Z. LSD 4.0: an improved database for comparative studies of leaf senescence. Mol. Horticulture. 2022; 2:24. 10.1186/s43897-022-00045-w PMC10515038 37789481

[21] Hua Z. , Tian D., Jiang C., Song S.H., Chen Z., Zhao Y., Jin Y., Huang L., Zhang Z., Yuan Y. Towards comprehensive integration and curation of chloroplast genomes. Plant Biotechnol. J. 2022; 20:12. 10.1111/pbi.13923 PMC9674321 36069606

[22] Jiang S. , Qian Q., Zhu T., Zong W., Shang Y., Jin T., Zhang Y., Chen M., Wu Z., Chu Y. et al. Cell Taxonomy: a curated repository of cell types with multifaceted characterization. Nucleic Acids Res. 2023; 51:D853–D860. 36161321 10.1093/nar/gkac816 PMC9825571

[23] Arita M. , Karsch-Mizrachi I., Cochrane G. The international nucleotide sequence database collaboration. Nucleic Acids Res. 2021; 49:D121–D124. 33166387 10.1093/nar/gkaa967 PMC7778961

[24] Leinonen R. , Sugawara H., Shumway M. International Nucleotide Sequence Database, C. The sequence read archive. Nucleic Acids Res. 2011; 39:D19–D21. 21062823 10.1093/nar/gkq1019 PMC3013647

[25] Sayers E.W. , Cavanaugh M., Clark K., Pruitt K.D., Sherry S.T., Yankie L., Karsch-Mizrachi I. GenBank 2023 update. Nucleic Acids Res. 2023; 51:D141–D144. 36350640 10.1093/nar/gkac1012 PMC9825519

[26] Jin E. , ZhaoD., WuG., ZhuJ., WangZ., WeiZ., ZhangS., WangA., TangB., ChenX.et al. OBIA: an Open Biomedical Imaging Archive. Genomics Proteomics Bioinformatics. 2023; 10.1016/j.gpb.2023.09.003.37806555

[27] Cao Y. , Tian D., Tang Z., Liu X., Hu W., Zhang Z., Song S. OPIA: an open archive of plant images and related phenotypic traits. Nucleic Acids Res. 2023; 10.1093/nar/gkad975. PMC10767956 37930849

[28] Wang G. , W.S. X.Z., Qu H., Fang X., Bao Y CROST: a comprehensive repository of spatial transcriptomics. Nucleic Acids Res. 2024; 10.1093/nar/gkad782. PMC10773281 37791883

[29] Cao R. , Ling Y., Meng J., Jiang A., Luo R., He Q., Li A., Chen Y., Zhang Z., Liu F. et al. SMDB: a spatial multimodal data browser. Nucleic Acids Res. 2023; 51:W553–W559. 37216588 10.1093/nar/gkad413 PMC10320082

[30] Li C. , Qian Q., Yan C., Lu M., Li L., Li P., Fan Z., Lei W., Shang K., Wang P. et al. HervD Atlas: a curated knowledgebase of associations between Human endogenous retroviruses and diseases. Nucleic Acids Res. 2024; 10.1093/nar/gkad904. PMC10767980 37870452

[31] Li H. , Wu S., Li J., Xiong Z., Yang K., Ye W., Ren J., Wang Q., Xiong M., Zheng Z. et al. HALL: a comprehensive database for human aging and longevity studies. Nucleic Acids Res. 2024; 10.1093/nar/gkad880. PMC10767887 37870433

[32] Sun Y. , Zheng X., Wang G., Wang Y., Chen X., Sun J., Xiong Z., Zhang S., Wang T., Fan Z. et al. MACdb: a curated knowledgebase for metabolic associations across Human cancers. Mol. Cancer Res. 2023; 21:691–697. 37027007 10.1158/1541-7786.MCR-22-0909 PMC10320464

[33] Xu T. , Gao W., Zhu L., Chen W., Niu C., Yin W., Ma L., Zhu X., Ling Y., Gao S. et al. NAFLDkb: a knowledge base and platform for drug development against nonalcoholic fatty liver disease. J. Chem. Inf. Model. 2023; 10.1021/acs.jcim.3c00395. 37167092

[34] Zhao X. , Modur V., Carayannopoulos L.N., Laterza O.F. Biomarkers in pharmaceutical research. Clin. Chem. 2015; 61:1343–1353. 26408531 10.1373/clinchem.2014.231712

[35] Califf R.M. Biomarker definitions and their applications. Exp. Biol. Med. (Maywood). 2018; 243:213–221. 29405771 10.1177/1535370217750088 PMC5813875

[36] Lippi G. , Mattiuzzi C. The biomarker paradigm: between diagnostic efficiency and clinical efficacy. Pol. Arch. Med. Wewn. 2015; 125:282–288. 25764446 10.20452/pamw.2788

[37] Ahmad A. , Imran M., Ahsan H. Biomarkers as biomedical bioindicators: approaches and techniques for the detection, analysis, and validation of novel Biomarkers of diseases. Pharmaceutics. 2023; 15:6. 10.3390/pharmaceutics15061630 PMC10303887 37376078

[38] Wang Y. , Lin Y., Wu S., Sun J., Meng Y., Jin E., Kong D., Duan G., Bei S., Fan Z. et al. BioKA: a curated and integrated biomarker knowledgebase for animals. Nucleic Acids Res. 2024; 10.1093/nar/gkad873. PMC10767812 37843156

[39] Field Y. , Boyle E.A., Telis N., Gao Z., Gaulton K.J., Golan D., Yengo L., Rocheleau G., Froguel P., McCarthy M.I. et al. Detection of human adaptation during the past 2000 years. Science. 2016; 354:760–764. 27738015 10.1126/science.aag0776 PMC5182071

[40] Voight B.F. , Kudaravalli S., Wen X., Pritchard J.K. A map of recent positive selection in the human genome. PLoS Biol. 2006; 4:e72. 16494531 10.1371/journal.pbio.0040072 PMC1382018

[41] Zhang P. , Luo H., Li Y., Wang Y., Wang J., Zheng Y., Niu Y., Shi Y., Zhou H., Song T. et al. NyuWa genome resource: a deep whole-genome sequencing-based variation profile and reference panel for the Chinese population. Cell Rep. 2021; 37:110017. 34788621 10.1016/j.celrep.2021.110017

[42] Shi Y. , Niu Y., Zhang P., Luo H., Liu S., Zhang S., Wang J., Li Y., Liu X., Song T. et al. Characterization of genome-wide STR variation in 6487 human genomes. Nat. Commun. 2023; 14:2092. 37045857 10.1038/s41467-023-37690-8 PMC10097659

[43] Taliun D. , Harris D.N., Kessler M.D., Carlson J., Szpiech Z.A., Torres R., Taliun S.A.G., Corvelo A., Gogarten S.M., Kang H.M. et al. Sequencing of 53,831 diverse genomes from the NHLBI TOPMed Program. Nature. 2021; 590:290–299. 33568819 10.1038/s41586-021-03205-y PMC7875770

[44] Johnson K.E. , Voight B.F. Patterns of shared signatures of recent positive selection across human populations. Nat. Ecol. Evol. 2018; 2:713–720. 29459708 10.1038/s41559-018-0478-6 PMC5866773

[45] Lin S. , Wu S., Zhao W., Fang Z., Kang H., Liu X., Pan S., Yu F., Bao Y., Jia P TargetGene: a comprehensive database of cell-type-specific target genes for genetic variants. Nucleic Acids Res. 2024; 10.1093/nar/gkad901. PMC10767789 37870478

[46] Wang Y. , Ling Y., Gong J., Zhao X., Zhou H., Xie B., Lou H., Zhuang X., Jin L., Han K.I. et al. PGG.SV: a whole-genome-sequencing-based structural variant resource and data analysis platform. Nucleic Acids Res. 2023; 51:D1109–D1116. 36243989 10.1093/nar/gkac905 PMC9825616

[47] Liu Y. , Zhang Y., Liu X., Shen Y., Tian D., Yang X., Liu S., Ni L., Zhang Z., Song S. et al. SoyOmics: a deeply integrated database on soybean multi-omics. Mol. Plant. 2023; 16:794–797. 36950735 10.1016/j.molp.2023.03.011

[48] Miao W. , Song L., Ba S., Zhang L., Guan G., Zhang Z., Ning K. Protist 10,000 Genomes Project. Innovation (Camb). 2020; 1:100058. 34557722 10.1016/j.xinn.2020.100058 PMC8456420

[49] Yang S. , Zong W., Shi L., Li R., Ma Z., Ma S., Si J., Bao Y., Li R., Xie J. ( PPGR: a comprehensive perennial plant genomes and regulation database. Nucleic Acids Res. 2023; 10.1093/nar/gkad963. PMC10767873 37933857

[50] Chen T. , Chen X., Zhang S., Zhu J., Tang B., Wang A., Dong L., Zhang Z., Yu C., Sun Y. et al. The Genome Sequence Archive family: toward explosive data growth and diverse data types. Genomics Proteomics Bioinformatics. 2021; 19:578–583. 34400360 10.1016/j.gpb.2021.08.001 PMC9039563

[51] Wang Y. , Song F., Zhu J., Zhang S., Yang Y., Chen T., Tang B., Dong L., Ding N., Zhang Q. et al. GSA: genome Sequence Archive. Genomics Proteomics Bioinformatics. 2017; 15:14–18. 28387199 10.1016/j.gpb.2017.01.001 PMC5339404

[52] Ma L. , ZouD., LiuL., ShireenH., AbbasiA.A., BatemanA., XiaoJ., ZhaoW., BaoY., ZhangZ. Database commons: a catalog of worldwide biological databases. Genomics Proteomics Bioinformatics. 2022; 10.1016/j.gpb.2022.12.004.36572336

[53] Ma L. , Zhang Z. The contribution of databases towards understanding the universe of long non-coding RNAs. Nat. Rev. Mol. Cell Biol. 2023; 24:601–602. 37147495 10.1038/s41580-023-00612-z

[54] Chen M. , Ma Y., Wu S., Zheng X., Kang H., Sang J., Xu X., Hao L., Li Z., Gong Z. et al. Genome Warehouse: a public repository housing Genome-scale data. Genomics Proteomics Bioinformatics. 2021; 19:584–589. 34175476 10.1016/j.gpb.2021.04.001 PMC9039550

[55] Gong Z. , Zhu J.W., Li C.P., Jiang S., Ma L.N., Tang B.X., Zou D., Chen M.L., Sun Y.B., Song S.H. et al. An online coronavirus analysis platform from the National Genomics Data Center. Zool Res. 2020; 41:705–708. 33045776 10.24272/j.issn.2095-8137.2020.065 PMC7671910

[56] Song S.H. , Ma L., Zou D., Tian D., Li C., Zhu J., Chen M., Wang A., Ma Y., Li M. et al. The global landscape of SARS-CoV-2 genomes, variants, and haplotypes in 2019nCoVR. Genomics Proteomics Bioinformatics. 2020; 18:749–759. 33704069 10.1016/j.gpb.2020.09.001 PMC7836967

[57] Zhao W.M. , Song S.H., Chen M.L., Zou D., Ma L.N., Ma Y.K., Li R.J., Hao L.L., Li C.P., Tian D.M. et al. The 2019 novel coronavirus resource. Yi Chuan. 2020; 42:212–221. 32102777 10.16288/j.yczz.20-030

[58] Li C. , MaL., ZouD., ZhangR., BaiX., LiL., WuG., HuangT., ZhaoW., JinE.et al. RCoV19: A One-stop Hub for SARS-CoV-2 Genome Data Integration, Variant Monitoring, and Risk Pre-warning. Genomics Proteomics Bioinformatics. 2023; 10.1016/j.gpb.2023.10.004.37898309

[59] Zhang Y.S. , Zou D., Zhu T.T., Xu T.Y., Chen M., Niu G.Y., Zong W.T., Pan R., Jing W., Sang J. et al. Gene Expression Nebulas (GEN): a comprehensive data portal integrating transcriptomic profiles across multiple species at both bulk and single-cell levels. Nucleic Acids Res. 2022; 50:D1016–D1024. 34591957 10.1093/nar/gkab878 PMC8728231

[60] Niu G. , Zou D., Li M., Zhang Y., Sang J., Xia L., Li M., Liu L., Cao J., Zhang Y. et al. Editome Disease Knowledgebase (EDK): a curated knowledgebase of editome-disease associations in human. Nucleic Acids Res. 2019; 47:D78–D83. 30357418 10.1093/nar/gky958 PMC6323952

[61] Xiong Z. , Yang F., Li M., Ma Y., Zhao W., Wang G., Li Z., Zheng X., Zou D., Zong W. et al. EWAS Open Platform: integrated data, knowledge and toolkit for epigenome-wide association study. Nucleic Acids Res. 2022; 50:D1004–D1009. 34718752 10.1093/nar/gkab972 PMC8728289

[62] Xiong Z. , Li M., Yang F., Ma Y., Sang J., Li R., Li Z., Zhang Z., Bao Y. EWAS Data Hub: a resource of DNA methylation array data and metadata. Nucleic Acids Res. 2020; 48:D890–D895. 31584095 10.1093/nar/gkz840 PMC6943079

[63] Li M. , Zou D., Li Z., Gao R., Sang J., Zhang Y., Li R., Xia L., Zhang T., Niu G. et al. EWAS Atlas: a curated knowledgebase of epigenome-wide association studies. Nucleic Acids Res. 2019; 47:D983–D988. 30364969 10.1093/nar/gky1027 PMC6324068

[64] Xiong Z. , Li M., Ma Y., Li R., Bao Y. GMQN: a reference-based method for correcting batch effects and probe bias in HumanMethylation BeadChip. Front. Genet. 2021; 12:810985. 35069703 10.3389/fgene.2021.810985 PMC8777061

[65] Zhao Y. , Wang J., Liang F., Liu Y., Wang Q., Zhang H., Jiang M., Zhang Z., Zhao W., Bao Y. et al. NucMap: a database of genome-wide nucleosome positioning map across species. Nucleic Acids Res. 2019; 47:D163–D169. 30335176 10.1093/nar/gky980 PMC6323900

[66] Zhang M. , Zong W., Zou D., Wang G., Zhao W., Yang F., Wu S., Zhang X., Guo X., Ma Y. et al. MethBank 4.0: an updated database of DNA methylation across a variety of species. Nucleic Acids Res. 2023; 51:D208–D216. 36318250 10.1093/nar/gkac969 PMC9825483

[67] Li R. , Liang F., Li M., Zou D., Sun S., Zhao Y., Zhao W., Bao Y., Xiao J., Zhang Z. MethBank 3.0: a database of DNA methylomes across a variety of species. Nucleic Acids Res. 2018; 46:D288–D295. 29161430 10.1093/nar/gkx1139 PMC5753180

[68] Zou D. , Sun S., Li R., Liu J., Zhang J., Zhang Z. MethBank: a database integrating next-generation sequencing single-base-resolution DNA methylation programming data. Nucleic Acids Res. 2015; 43:D54–D58. 25294826 10.1093/nar/gku920 PMC4384011

[69] Kang H. , Huang T., Duan G., Meng Y., Chen X., He S., Xia Z., Zhou X., Chao J., Tang B. et al. TCOD: an integrated resource for tropical crops. Nucleic Acids Res. 2024; 10.1093/nar/gkad870. PMC10767838 37843152

[70] Schuler G.D. , Epstein J.A., Ohkawa H., Kans J.A. Entrez: molecular biology database and retrieval system. Methods Enzymol. 1996; 266:141–162. 8743683 10.1016/s0076-6879(96)66012-1

[71] Madeira F. , Pearce M., Tivey A.R.N., Basutkar P., Lee J., Edbali O., Madhusoodanan N., Kolesnikov A., Lopez R. Search and sequence analysis tools services from EMBL-EBI in 2022. Nucleic Acids Res. 2022; 50:W276–W279. 35412617 10.1093/nar/gkac240 PMC9252731

